# Particle‐Based Tracking of Cold Pool Gust Fronts

**DOI:** 10.1029/2019MS001910

**Published:** 2020-05-04

**Authors:** Olga Henneberg, Bettina Meyer, Jan O. Haerter

**Affiliations:** ^1^ Niels Bohr Institute University of Copenhagen Copenhagen Denmark

**Keywords:** cold pools, tracking, convection, self‐organization, precipitation, large eddy simulations

## Abstract

The gust fronts of convective cold pools (CPs) are increasingly recognized as loci of enhanced triggering for subsequent convective cells. It has so far been difficult to track these gust fronts in high‐resolution data, such as large eddy simulations (LES)—rendering mechanistic analysis of CP interaction incomplete. Here, a simple tracking method is defined, tested, and applied, which uses horizontal advection and a condition on horizontal divergence, to emit tracers at the perimeter of surface precipitation patches. Tracers are then reliably transported to the gust front, yielding closed bands marking the CP boundary. The method thereby allows analysis of the dynamics also *along* the gust front, which allows to identify point‐like loci of pronounced updrafts. The tracking works well for a single idealized CP and reliably tracks a population of CPs in a midlatitude diurnal cycle. As the method uniquely links CPs and their tracers to a specific parent precipitation cell, it may be useful for the analysis of interactions in evolving CP populations.

## Key Points

- Horizontally advected tracers are suitable for the tracking of cold pools gust fronts
- The method allows for the analysis of radial and azimuthal dynamics
- Convergence hot spots with strong updrafts can be identified

## 1 Introduction

**Figure 1:**
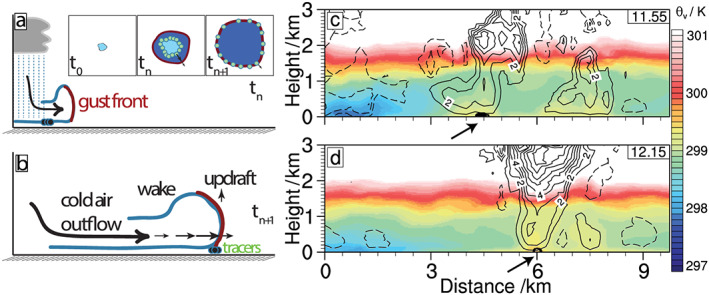
Schematic of cold pool dynamics and its tracking in the simulation. (a) An early stage of the gust front at time $t_{n}$ advancing from a precipitation event. Tracer particles emitted near the boundary of the surface precipitation area are shown as black filled circles. (b) Gust front at time $t_{n+1}>t_{n}$. Inset to (a): Near‐surface horizontal cross section, showing the onset of surface precipitation (time $t_{0}$), initial emission of tracer particles (time $t_{n}$); and gust front with tracer particles properly mapping the cold pool edge (time $t_{n+1}$). (c) Cross sections of virtual potential temperature $\theta_{v}$ (see color bar) overlaid by contour lines of vertical velocity (solid lines for positive, dashed lines for negative velocities, labels mark contours in units of m s ${}^{-1}$). Downdrafts (dashed lines) at distance 0–2 km generate a cold pool at low levels. Tracers are located near the gust front (solid lines), as shown by their position, projected onto the cross section (black circles, highlighted by an arrow). Note that the tracers are here located in locally buoyant regions, indicative of cold pool gust fronts at a later stage of their life cycle. (d) Similar to (c), but at a later time, as marked in panel. Note that the two updraft areas have now merged and that updraft speed has increased.

Precipitation from cumulus clouds can form cold and dry downbursts by the evaporative cooling of precipitation (Doswell, 2001). The resulting volume of air, colder and denser than the ambient atmosphere, is often referred to as a *cold pool* (CP). When reaching the surface, this CP spreads radially as a gravity current. Gravity currents have been studied for decades, both theoretically (Benjamin, 1968; Von Karman, 1940), in tank experiments (Keulegan, 1957; Kuenen, 1950) and in Earth's atmosphere (Byers & Braham, 1949; Webster & Lukas, 1992). The CP gravity current is confined by a leading gust front, typically several hundred meters high, which can advance radially at speeds of ∼4 m s ${}^{-1}$ for weak tropical CPs (Drager & van den Heever, 2017; Zuidema et al., 2017). At the gust front air is deflected into the vertical and partially mixed back into the CP interior, at a region termed the *CP wake*(Benjamin, 1968) (Figures 1a and 1b).

CPs can emerge from various types of convective clouds: In shallow cumuli, often found in trade wind regions, precipitation is weak (0.5–2 mm day ${}^{-1}$) and thus CPs of modest momentum are formed (Wang & Feingold, 2009); CPs resulting from deep convective precipitation in the tropics (Tompkins, 2001a, 2001b; Zuidema et al., 2017), or during midlatitude summer (Haerter et al., 2019; Leutwyler et al., 2016; Rotunno et al., 1988), tend to be much larger and carry substantially greater momentum due to more intense precipitation up to 50 mm hr ${}^{-1}$ (Westra et al., 2014). We are here interested in continental convection, where the current method can help understand convective organization and the occurrence of flash flooding.

CPs can generally be characterized as constituting a “footprint” of the parent precipitation cell: Convection is inhibited in the CP interior, whereas further outward buoyancy is often enhanced. The inhibition results from two stabilizing effects: evaporative cooling, typically 2–3 K, and subsidence drying, typically 0.3–1 g kg ${}^{-1}$ (Young et al., 1995), due to the associated downdrafts. These stable conditions, which dominate the subcloud atmosphere beneath the precipitation event and a few kilometers away from it (Drager & van den Heever, 2017), suppress further convective activity (Emanuel, 1989) until the air mass has recovered. In contrast, the CP gust front is characterized by increased convective activity as a result of increased buoyancy and strong horizontal winds that induce a band of near‐surface convergence ahead of the CP gust front. Due to conservation of mass, this circulation generates strong vertical updrafts that favor the triggering of new convection events, often referred to as *mechanical forcing* (Droegemeier & Wilhelmson, 1985; Thorpe et al., 1982). In addition, enhanced moisture in this area favors *thermodynamical triggering* of convection by the destabilization of the atmosphere (Feng et al., 2015; Tompkins, 2001a, 2001b). Moisture can originate from increased surface latent heat fluxes through the strong winds near the gust front (Langhans & Romps, 2015), advection of environmental moisture (Schlemmer & Hohenegger, 2016; Seifert et al., 2015), and the recycling of evaporated precipitation (Langhans & Romps, 2015; Tompkins, 2001a).

The redistribution of moisture and temperature by CPs, combined with the mechanical forcing, acts to structure the cloud field (Bretherton & Blossey, 2017; Haerter, 2019; Jeevanjee & Romps, 2013; Schlemmer & Hohenegger, 2014; Wang & Feingold, 2009). In diurnal cycle simulations, such organization often leads to increasing spatial scales, which appear to be associated with increasing precipitation rates (Haerter et al., 2017; Jeevanjee & Romps, 2013; Khairoutdinov & Randall, 2006; Schlemmer & Hohenegger, 2014; Wang & Feingold, 2009). Schlemmer and Hohenegger (2014) describe a positive feedback of increased precipitation rates and larger patches of moist air that allow clouds to deepen through reduced entrainment (Kuang & Bretherton, 2006; Stirling & Stratton, 2012; Yeo & Romps, 2012). We here focus on such diurnal effects and the method presented may help explore mechanical interaction effects as they are found within the continental diurnal cycle.

Previous studies seek to disentangle dynamical triggering by strong updrafts at the gust front and thermodynamic triggering by enhanced buoyancy due to increased moisture (Feng et al., 2015; Jeevanjee & Romps, 2015; Torri et al., 2015). For this purpose, it is necessary to locate the CP gust front in observational and numerical data. Buoyancy anomalies have been used in previous efforts aimed at identifying CPs and their boundaries, such as Schlemmer and Hohenegger (2016), who used a drop in integrated virtual potential temperature or Tompkins (2001a) and Feng et al. (2015), who applied a buoyancy threshold. Drager and van den Heever (2017) used radial derivatives and identified the steepest gradient of buoyancy, rather than a finite threshold, as a less ambiguous criterion. Triggering of subsequent convection, however, often occurs near the narrow convergence bands (Figures 1c and 1d), which need not coincide with any thermodynamic measure. Indeed, when CPs age, their initial temperature depression gradually equilibrates (Grant & van den Heever, 2016; Szoeke et al., 2017; Torri & Kuang, 2016) and virtual potential temperature may even increase at the gust front (Fournier & Haerter, 2019; Tompkins, 2001a). Thus, the area of CPs identified by temperature‐based methods tends to reduce during later stages of the CP lifetime (Feng et al., 2015). This retreating feature stands in contradiction to the dynamics of CP gust fronts, which often continue to spread by inertia, even if the temperature anomaly is reduced (Fournier & Haerter, 2019; Grant & van den Heever, 2018). Thus, if we are interested in the mechanical triggering of convective triggering, it is useful to define the CP as the area confined by the band of strong horizontal convergence near the surface. CP gust fronts have previously been identified based on the wind field, using a radial gradient‐based method (Fournier & Haerter, 2019). In the following, we refer to the region of the convergence peak as the *CP edge*. As this dynamically defined CP edge will always lie ahead (or on top) of the thermodynamic boundary, this method has the advantage that both types of convective triggering occur within the identified CP area.

**Figure 2:**
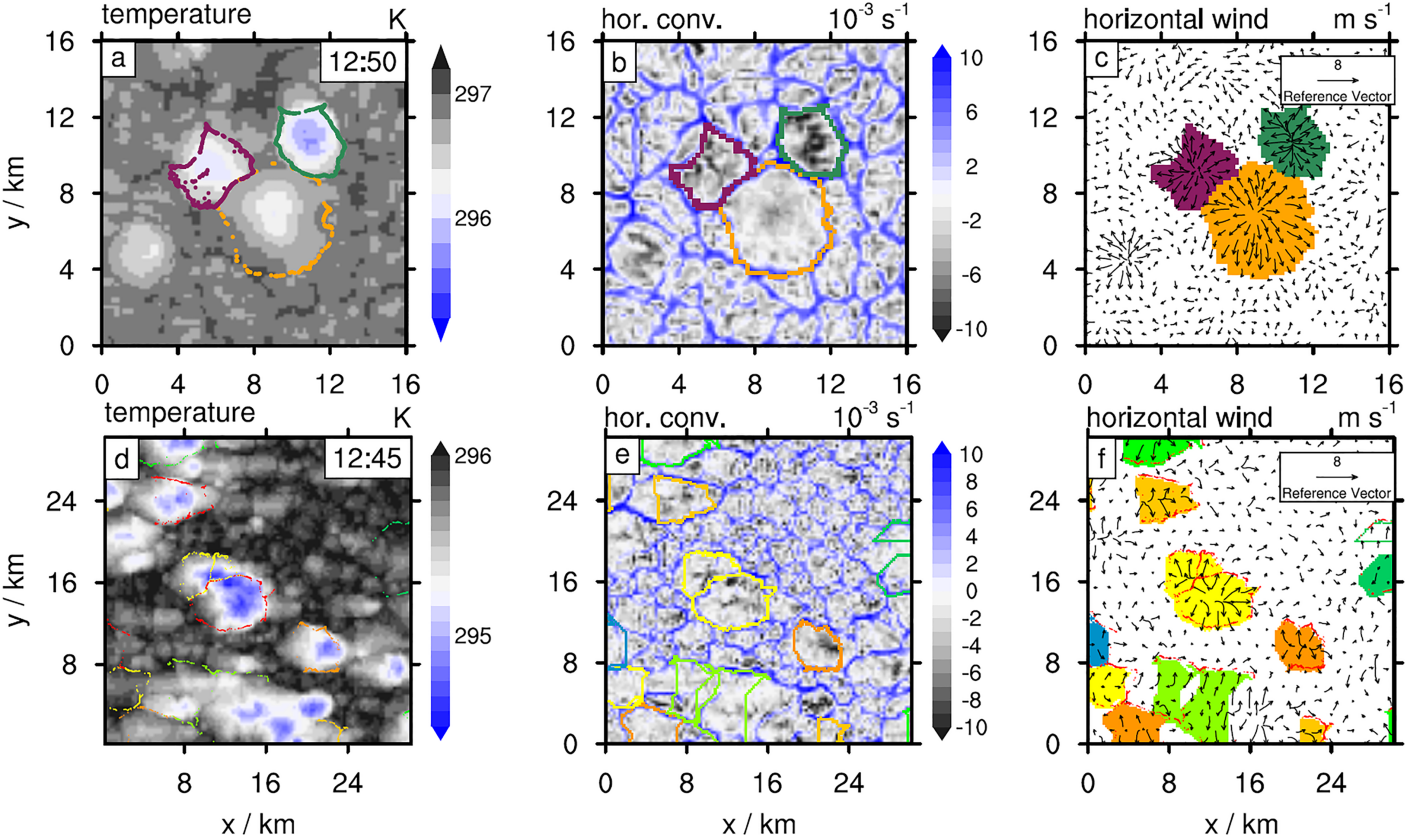
Particle‐based CP tracking with and without large‐scale wind shear. (a–c) Horizontal cross sections for the reference simulation without wind shear (CTRL) for the lowest model level ( z=50 m) at time as labeled in panel (a). (a) Temperature field (blue and gray shades, see color bar) and tracer positions for three CPs (purple, orange, and green points). (b) Horizontal convergence field (gray and blue shades, see color bar, units $10^{3}$ s ${}^{-1}$), showing grid cells occupied by tracers as well as additional grid cells filled in between tracers (*Details:* section 3). (c) Horizontal wind field and CP objects. Note the maroon‐colored CP, which appears indented toward to top left—a result of early‐stage tracers occasionally having to “catch up” with the gust front. (d–f) Similar to panels (a–c) but for a simulation with large‐scale advection (ADV). Note, however, that the resultant tracer density for ADV is more anisotropic and that our interpolation method can occasionally cause poor approximations, where CP boundaries intersect with the actual CP area (e.g., for the orange contours, shown in panel e). Improvements, left for a separate study, could be obtained by increasing the tracer number N (see Table 1).

As in Haerter et al. (2019), the present tracking method exploits the fact that CPs generally are confined by a band of convergence, using Lagrangian tracers. Allowing advection in all three spatial dimensions, Lagrangian particles were previously applied to analyze CPs (Grant et al., 2018; Torri & Kuang, 2016; Torri et al., 2015). To prevent mixing of particles into the upper boundary layer, the current particle‐based method demands that tracer particles are constrained to travel only horizontally (Figure 1a,b). Under this constraint, tracers robustly detect the often rather diffuse convergence structure that surrounds a precipitation event and uniquely associates it with its parent precipitation event (Figure 2b), even for a dense population of cells.

Haerter et al. (2019) employed radial advection of tracers, emitted at the circumference of surface rain objects, to map out the gust front of each CP. The method here builds on that in Haerter et al. (2019) by allowing each tracer to follow the local two‐dimensional flow, which opens for analysis of the internal front dynamics, such as tangential flow along the gust front. The current method further adds a clearly defined tracer emission time. Additionally, our criterion on tracer emission, which requires a sufficient divergence under rain cells, allows for tracers to properly map out the CP gust front to all sides of the parent rain event. The method further outputs additional tracer information, such as the detection of the entire CP object, rather than just its edge. We here provide a thorough investigation of the tracer dynamics, and discuss within an idealized case, why and under which circumstances the tracer method works well in identifying gust fronts.

We here also deal with merging and splitting parent precipitation cells, which was not done previously. By incorporating a consistent handling of such events, the CPs gust fronts of merging rain cells are also merged. Finally, the method employs a criterion for termination of each CP track. The tracking is successfully tested on large eddy simulation (LES) data containing strongly interacting and colliding CPs. Interacting CPs complicate detection, because CPs do not appear in spatial isolation from one another any longer.

The simulations, used to develop the particle‐based tracking, are described in section 2. In section 3 the details of the methodology are explained. Finally, the performance of the tracking in LES is assessed (section 4) and statistical behavior of the identified CPs discussed. We close with an outlook on possible applications (section 5).

## 2 LES Data

The present study addresses dynamical lifting near the CP gust front, and therefore focuses on continental diurnal cycle dynamics, where forced lifting has previously been shown to dominate (Haerter et al., 2019; Moseley et al., 2016). The method is applied and tested on data from an LES of idealized deep convection occurring during a diurnal cycle over a wet land surface. For this purpose, the University of California, Los Angeles, Large Eddy Simulator (UCLA‐LES) (Stevens et al., 1999, 2005) is run for conditions typical of midlatitude summer days with convection, similar to previous simulations by Moseley et al. (2016, 2019) and Haerter et al. (2017). This simulation is considered well‐suited as a test bed for the particle‐based tracking because the forced convection generates CPs that strongly interact and collide. Furthermore, due to the pronounced diurnal cycle, the CPs that emerge at different stages of the simulations vary substantially in terms of the associated temperature anomalies, life cycle, and spreading velocities. This allows to test the tracking on a wide spectrum of CPs.

The horizontal resolution is dx=dy=200 m and the vertical resolution in the lowest 1 km is 100 m, extending to 200–400 m over 75 vertical levels up to the domain height of 16.8 km. An area of (204.8 km)^2^ (1,024 × 1,024 grid points) is simulated with double‐periodic lateral boundary conditions. The simulation uses an adaptive time step on the order of 1 to 5 s and one model day was simulated. The model output time interval was set to 5 min. The surface is taken to have infinite heat capacity, that is, it does not respond to downwelling radiation. Deep convection is strongly forced by prescribed, sinusoidally varying, diurnal surface temperature of mean 23 °C and an amplitude of 10 K, and interactive surface evaporation, modeled as 70% compared to that of a water surface of equal temperature. Subgrid‐scale turbulence is parameterized by the Smagorinsky scheme (Smagorinsky, 1963). Cloud microphysical processes are parameterized within the two‐moment cloud microphysics scheme following Seifert and Beheng (2006). The simulation is initialized using vertical temperature and moisture profiles as in Moseley et al. (2016) and Haerter and Schlemmer (2018).

In the course of diurnal heating, the simulated cloud field transitions from shallow cumulus to deep cumulonimbus convection around noon (12 hr). Domain mean precipitation intensity initially increases and eventually peaks around 15 hr (Haerter et al., 2017) as convection deepens and individual cells intensify. Thereafter, the number of precipitation cells decreases while individual cells often intensify further. In addition to the shear‐free simulation described above (CTRL), the performance of the tracking was also tested on a simulation with horizontal advection (ADV). This additional simulation uses prescribed large‐scale forcing in the x direction with a wind speed profile that increases linearly from u=0 m s ${}^{-1}$ at the surface to u=10 m s ${}^{-1}$ at z=10 km. ADV mimics large‐scale advection as could be realistic for midlatitude conditions (*Details:* Moseley et al., 2016).

## 3 Methodology

The particle‐based CP tracking is suitably described in terms of the life cycle of a CP: the identification of the parent precipitation event (section 3.1), the timing (section 3.2) and location (section 3.3) of tracer particle seeding, as well as the the details of tracer advection (section 3.4). We finally describe, how the CP object is identified (section 3.5) and at which time a CP object is considered terminated (section 3.6). It is then discussed, using an idealized case, why and under which circumstances the tracking works (section 3.7).

### 3.1 Identification of Parent Precipitation Tracks

Precipitation cells are tracked by identifying the spatiotemporal overlap of objects using an iterative rain cell tracking (IRT) (Moseley et al., 2013, 2019), which assigns a unique track identifier (ID) to each precipitation track. Since the focus of this study is on deep convection, rain from shallow convection and drizzle is disregarded by imposing a threshold of $I_{0}=1$ mm hr ${}^{-1}$ to identify the surface precipitation objects (Table 1). When two tracks merge, the resulting merged object receives the track ID corresponding to the previously larger object, while the smaller one terminates. The CP gust fronts, corresponding to the merging rain tracks, are handled analogously. We find this to be reasonable, as the effect of rain objects merging is to force air that is captured in between the previously disparate rain objects to be pushed out laterally—leaving two gust front contours behind, that are no longer closed. By also merging these, a longer gust front contour, surrounding the now merged rain objects, is obtained. When a track splits, the largest fragment keeps the previous track ID, while all smaller fragments are subsequently given a distinct, new track ID. Fragmentation hence constitutes the inverse of a merging event: Previously connected CP gust front contours must now be considered as two.

**Table 1:** Parameter Summary

Parameter	Symbol	Value used
Precipitation cell tracking (IRT)		
Threshold surface precipitation rate	$I_{0}$	1 mm hr ${}^{-1}$
Threshold precipitation object area	$A_{0}$	50 grid cells
Particle‐based cold pool tracking		
Number of tracers per cold pool	N	500
Divergence threshold	$D_{0}$	2.5 $\times 10^{-3}$ s ${}^{-1}$
Termination threshold	$f_{\text{end}}$	0.6

The present simulations are dominated by stationary precipitation cells, which terminate because CPs shut off their updrafts. Therefore, splitting precipitation tracks are most often fragments of a diminishing precipitation track, which do not cause an additional CP. Thus, precipitation tracks resulting from splitting are not considered to potentially form another CP in this study. The IRT demands a minimum surface precipitation object area $A_{0}$. To initialize the tracers at a reasonable CP size, $A_{0}$ is here set to $A_{0}=50$ grid cells (2 km ${}^{2}$). The advantage of the tracer method is its low sensitivity to the thresholds $I_{0}$ and $A_{0}$, because tracers are quickly advected away from their initial position toward the convergence (*Details*: section 3.7). Hence, neither the exact starting time of the precipitation track nor its precise shape will influence the shape of the CP object.

### 3.2 Timing of Tracer Particle Emission

**Figure 3:**
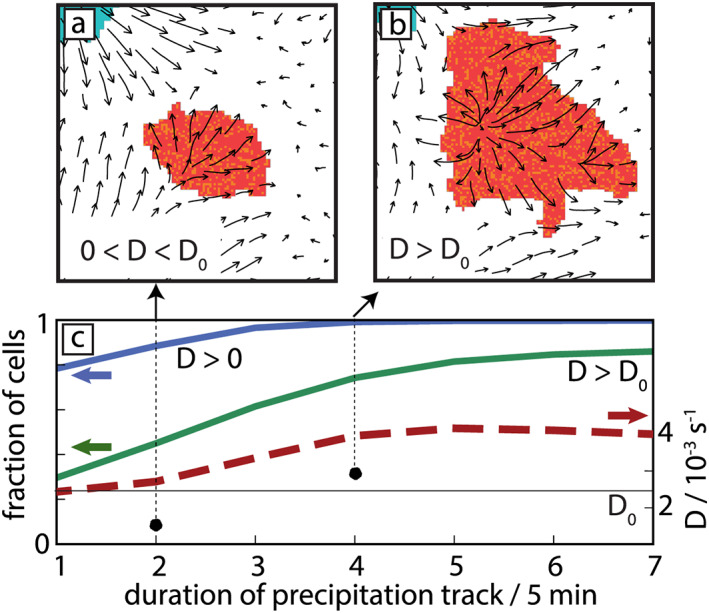
Divergence criterion for tracer initialization. (a) Schematic adapted from a simulation without imposed large‐scale advection, showing the near‐surface horizontal wind field with a superimposed precipitation object (red shading). The divergence is positive but below the threshold $D_{0}$ (marked by a black point and arrow in panel (c). (b) Similar to (a), but 10 min later. Note that now $D>D_{0}$. (c) Fraction of precipitation cells with average horizontal divergence ( D>0, blue curve) and divergence $D>D_{0}$ (green curve, $D_{0}$ marked by a thin horizontal line) across the area of the precipitation object. Average values over all precipitation objects with D>0 (dashed red curve).

Some caution is required as to when to start the trajectories of the tracers after the identification of the precipitation track, since a delay occurs between the time when air is evaporatively cooled and the time when it reaches the surface to spread laterally. The choice of the minimum precipitation track area, *A*_0_, already rules out that CPs start at a very early development stage of the precipitation cell. However, tracers should not be placed before the CP has developed a spreading wind field of similar strength as the preexisting wind field, which may, for example, be caused by other CPs. $A_{0}$. The example of a late stage of the diurnal cycle (Figure 3), where relatively strong preexisting winds near the surface from older CPs are often present, illustrates the sensitivity of the CP tracking to the tracer emission time: tracers emitted at the boundary of the surface precipitation patch (colored in red in Figure 3a) will all be advected towards the top‐right by the background wind and thus will not properly surround the CP. Ten minutes later (Figure 3b), the CP has generated its own wind field, which properly distributes the tracers along the convergence band surrounding the CP. To take this delay into account, we demand sufficient average horizontal divergence within the area of the precipitation object, before tracers are emitted and their trajectories initialized. The average horizontal divergence D for a given precipitation object is computed as

(1) $$D=\bar{\nabla_{h}\cdot {\vec{v}}_{h}}\;,$$

where ${\vec{v}}_{h}\equiv (u,v)$ is the horizontal wind vector, $\nabla_{h}\equiv (\partial_{x},\partial_{y})$, and the overline denotes the spatial average over the area of the surface precipitation object. To compute D from the numerical simulation output, we approximate the horizontal divergence within each model gridbox (i,j,k) as

(2) $$D_{i,j,k}\equiv (u_{i+\frac{1}{2},j,k}-u_{i-\frac{1}{2},j,k})/dx+(v_{i,j+\frac{1}{2},k}-v_{i,j-\frac{1}{2},k})/dy\;,$$

where i,j, and k denote the indexes of the gridbox center and $u_{i\mp \frac{1}{2},j,k}$ denotes the velocity in x direction at the left and right cell boundary (an analogous definition applies for v and the index j). For any precipitation object, the average in equation (1) is then computed by summing all contributions within the object area A and dividing by S, the total number of grid cells in A, namely

(3) $$D=\underset{(i,j)\in A}{\sum }D_{i,j,k}/S\;,$$

where we choose k to denote the lowest model level, located at z=50m. The majority of precipitation tracks show divergence ( D>0) already at the first time step of the track and even those with initial convergence ( D<0) mostly develop D>0 after few time steps (Figure 3c). Averaging separately over all tracks with D>0 and D<0 shows that the divergence generated by the spreading CP typically far exceeds the residual convergence that was present before the CP formed (Figure 3c). On average, CPs have D≈2.5 $\times 10^{-3}$  s ${}^{-1}$ in the first time step, a value which increases subsequently. We use this mean value $D_{0}\equiv$ 2.5 $\times 10^{-3}$ s ${}^{-1}$ as a threshold that needs to be exceeded before tracers are emitted. The accumulated number of tracks which have a diverging wind field above this threshold (Figure 3c, green line) shows that most tracks reach this value within the first five time steps.

In the presence of large‐scale advection (ADV), precipitation tracks show a similar diverging wind field at the first time step with an averaged value around $D_{0}$, as the background wind is small near the surface (∼0.05 m s^−1^) and only increases aloft (Figures 2d and 2e). We caution, however, that a diverging wind field does not always guarantee that the wind can distribute the tracers around the center if background winds exceed the spreading velocity of the CP.

### 3.3 Location of Tracer Particle Emission

Once the divergence field of the precipitation object exceeds the threshold *D*_0_, the tracers are initialized at the boundary grid cells of the identified precipitation object in the lowest model level at z=50 m. A boundary grid cell is a grid cell with at least one of its eight neighboring cells not belonging to the precipitation object. The lowest model level is chosen because the surface represents the solid boundary that forces the cold air to spread laterally. The convergence pattern produced is thus most distinct at the surface. All tracers placed around the same precipitation object are assigned a common CP ID, identical to the associated precipitation track ID. Furthermore, each individual tracer particle receives a unique tracer ID. To avoid biases in mean tracer statistics, as, for example, the CP radius, each CP is tracked by the same number N of tracers irrespective of the circumference and shape of the precipitation object and the time when tracers are started (Table 1). This means, that for precipitation objects with large area, the count of boundary grid cells exceeds the number of tracer particles to be placed. Hence, not at every boundary grid cell tracers will start a trajectory. To nonetheless ensure tracer particles to be distributed relatively evenly around the CP, they are placed in random order at the centers of the boundary grid cells. If efficiency in postprocessing is not critical, also a larger number of tracers could be emitted to allow for better resolution of the CP contour. For precipitation objects with small areas the number of tracers to be placed, N, sometimes exceeds the count of boundary grid cells. After placing one tracer in each of these boundary grid cells, the remaining tracers are placed at random within any boundary grid cell already occupied, however, at randomly perturbed locations within the respective grid cells. This procedure is repeated for additional seeding within doubly occupied grid cells, if necessary, until all N tracers have finally been placed. Tracer particles are advected at the same time step at which they are placed, using the wind field present at that time step (section 3.4).

### 3.4 Tracer Particle Advection

Tracking CPs using horizontally advected tracers builds on the consideration that tracers will become trapped in the narrow bands of horizontal convergence, that is, the gust fronts, that typically surround the CPs (Figure 2). Since the tracers cannot move vertically, they must move along with the expanding CP boundary. One must be aware that the tracer concentration does not correspond to the concentration of an actual atmospheric tracer, as, for example, aerosols or dust, for which mass conservation does not allow for the concentration increase of purely passive tracers. Such physical particles would be lifted vertically as soon as they enter the convergence zone. This illustrates that a high concentration of tracking tracers reflects the presence of strong updrafts aloft.

The horizontal velocity field within the lowest model level is interpolated bilinearly from the model grid to the tracer positions. At every time step, $t_{i}$, each tracer is advected by a simple Euler scheme during the time interval Δt to its new position $x(t_{i+1})$, where $t_{i+1}\equiv t_{i}+\Delta t$, using the spatially bilinearly interpolated velocities, $u(x(t_{i}),t_{i})$, at their current location $x(t_{i})$:

(4) $$x(t_{i+1})=x(t_{i})+\Delta t\;u(x(t_{i}),t_{i}).$$

To achieve an improved approximation to the advected particle position, updated tracer positions are calculated from iterating over n sub–time steps $t_{j}\in \{t_{i},t_{i}+\Delta t/n,\ldots ,t_{i}+(n-1)\Delta t/n\}$ and $t_{j+1}\equiv t_{j}+\Delta t/n$, as

(5) $$x(t_{j+1})=x(t_{j})+\frac{\Delta t}{n}\;u(x(t_{j}),t_{i})\;.$$

For the model data with a resolution of dx=dy=200 m and output time step of 5 min, it was found that computing particle positions and velocities once every minute, thus n=5, rather than with Δt=5 min (the model output interval) gave a sufficient improvement of tracer positions. For other resolutions a possible benefit of an additional temporal interpolation between output time steps should be considered. Figures 1c and 1d show a qualitative example of tracer positions in a horizontal‐vertical cross section.

Specifically for high‐resolution simulations (section 3.7) at comparably low output time step, the choice of iteration time step has an effect on the exact position of the tracers relative to the convergence band and thus the quantitative result for the CP radius. However, the propagation speed of the CP gust front and the finding that tracers accumulate near locations of strong convergence are not affected by the choice of *n*.

### 3.5 Identifying the CP Object

**Figure 4:**
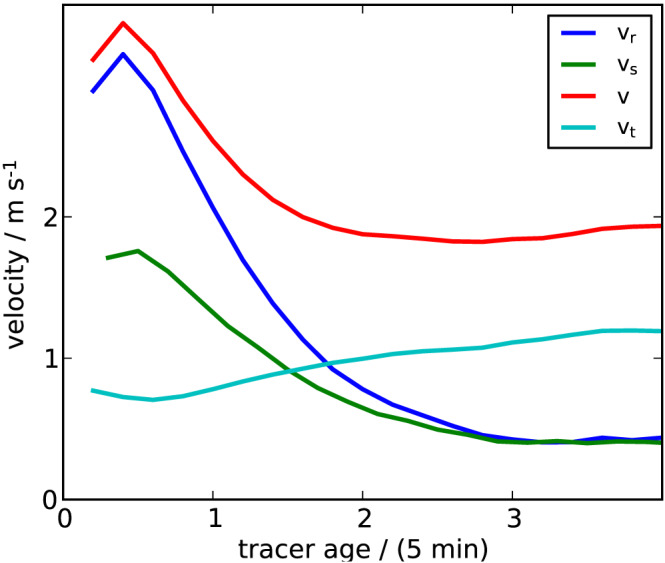
Tracer velocity versus tracer age. Spreading velocity $v_{s}$ of the CP edge, calculated by the change in the distance of the tracers to the CP center,  and the local horizontal velocity field at the tracer positions (absolute velocity v), further decomposed into the radial velocity $v_{r}$ and tangential velocity $v_{t}$. The data represent averages over all CPs and tracers conditional on a given tracer age.

We define the *CP object* as the area enclosed by the tracer particles of equal CP ID. In most cases, the tracers do not form a connected path along the model grid. In particular, when CPs collide, tracers are often advected tangentially along the CP boundaries (Droegemeier & Wilhelmson, 1985). This can be seen in the time series of velocities at the CP edge (Figure 4): The velocity component tangential to the CP edge (*v_t_*) gradually increases with CP age, whereas the radial component (*v*_*r*_) strongly decreases. To fill the resulting gaps in between tracers (cf. Figures 2a and 2b) and obtain a closed contour, the position of each tracer i is first expressed in polar coordinates ( $r_{i}$, $\phi_{i}$) relative to the CP center. The CP center is assumed to be colocated with the center of gravity of the parent precipitation object. Tracers are now sorted by their respective angles $\phi_{i}$ in order to identify tracers of closest angular distance $\left|\phi_{i}-\phi_{i+1}\right|$. Looping through this sorted list, gaps are identified by first checking if consecutive tracers of closest angular distance are located within the same grid cell. If not, the gap between tracers i and i+1 is closed by a straight line connecting i and i+1. All grid cells crossed by this line and cells containing tracers are marked as CP boundaries. Once a closed circumference around the CP center is defined, the CP object is identified as the set of all grid cells that are part of the circumference or lie in its interior (Figures 2c and 2f). The area of the identified CP is determined as the number of grid cells that are part of the CP object and that are not occupied by a younger CP (one for which tracers were emitted at a later time).

### 3.6 Termination of CP Track

Termination of the CPs is detected by a reduction in the area of the CP object. The size of a CP is often reduced by intruding neighboring CPs or by new CPs forming along its front. CP object size is found to fluctuate very little over the course of time until it significantly reduces in size. As a criterion for the time of termination, we use the ratio

(6) $$f=\frac{A(t_{i})}{\max\{A(t_{n}),t_{n}\in [0,t_{i}]\}}\;,$$

where $t_{n}$ denotes all time steps at which tracers corresponding to a given CP are present, relative to the time of their emission. For any time step $t_{i}$, f hence measures the ratio between the current area of the CP, A(t), and its maximally reached area in the time interval $0\leq t_{n}\leq t_{i}$. As a criterion for termination, we demand that $f<f_{\text{end}}$, where $f_{\text{end}}\in [0,1]$ is an adjustable parameter. This definition allows CPs at different stages of convective organization to be captured on equal footing: In practice, CPs at early stages within the diurnal cycle grow to smaller areas and are “overrun” more quickly by others, while those at later stages have longer lifetimes. f takes this time dependence of maximum CP area into account by making track termination relative to each CPs intrinsic life cycle. We here found $f_{\text{end}}=0.6$ to constitute a reasonable choice (Table 1).

### 3.7 Proof of Concept

**Figure 5:**
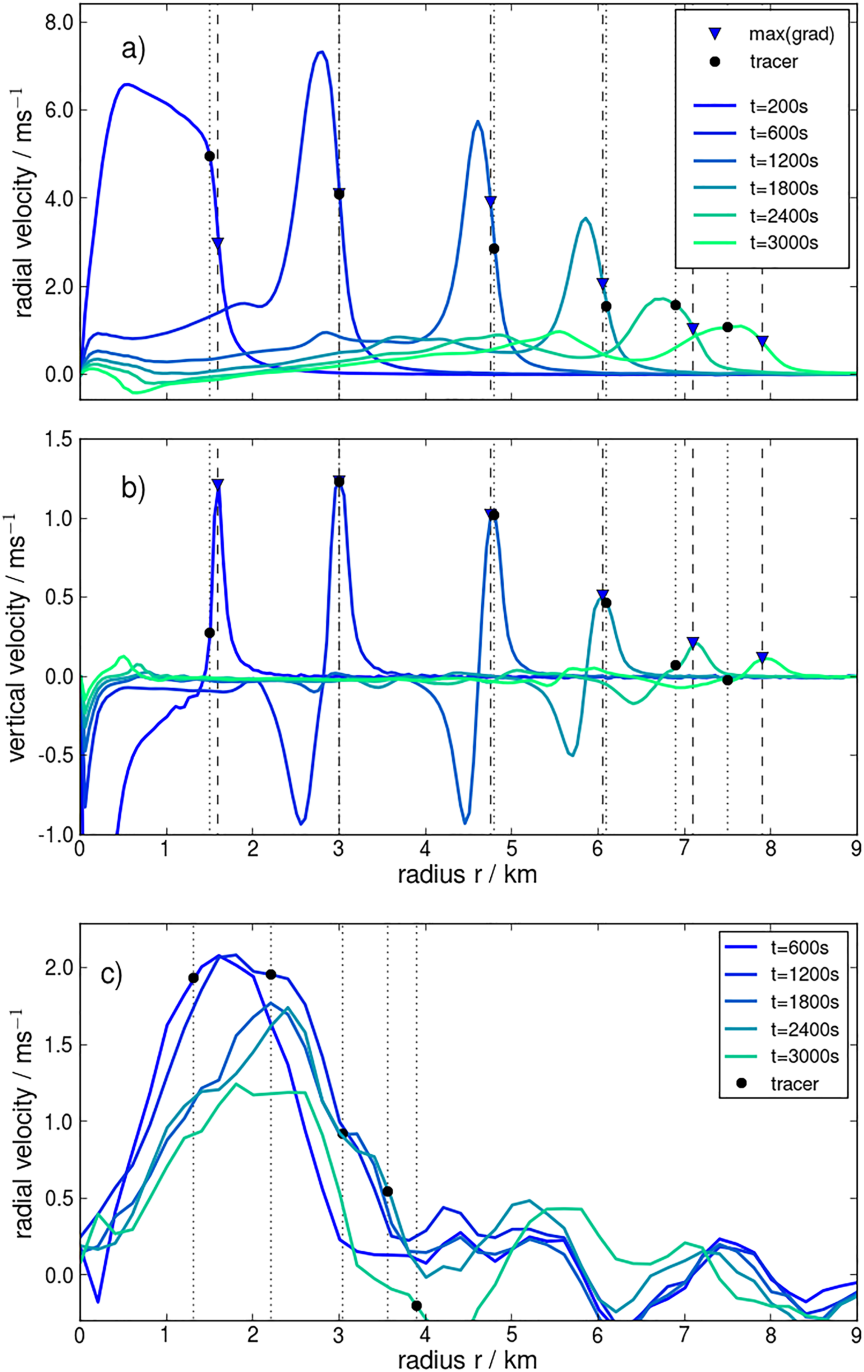
Average radial and vertical velocity and tracer position. (a) Azimuthally averaged radial profiles of the radial velocity $v_{r}(r)$ for an idealized CP (at times marked in legend). (b) Analogous to (a) but for vertical velocity w(r). (c) Similar to (a) but an example CP from the UCLA‐LES data (cf. Figure 10, orange). Times relative to tracer emission are marked in the legend. Black dots in all panels indicate the respective average tracer positions for each time point. Triangles in (a) and (b) mark the average radii of the convergence zone.

**Figure 6:**
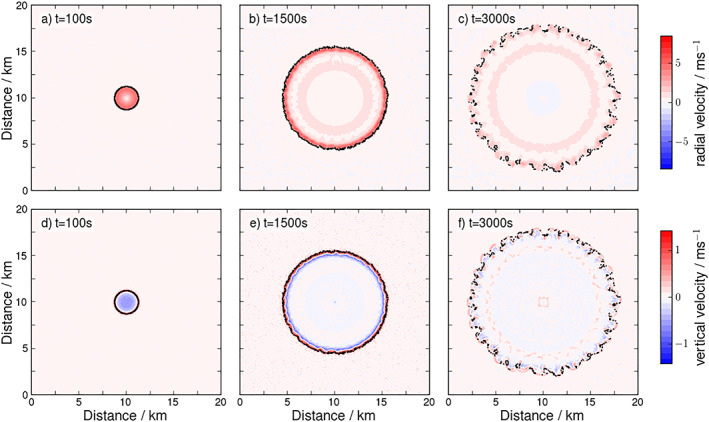
Tracer dynamics in a simplified flow. Time evolution of the CP in terms of the radial velocity (top: a–c) and vertical velocity (bottom: d–f) at the lowest model level at different times (as marked in panels). The tracers (black dots) first are homogeneously distributed along the CP rim, before they eventually accumulate in the clefts (see t=3,000 s).

**Figure A1:**
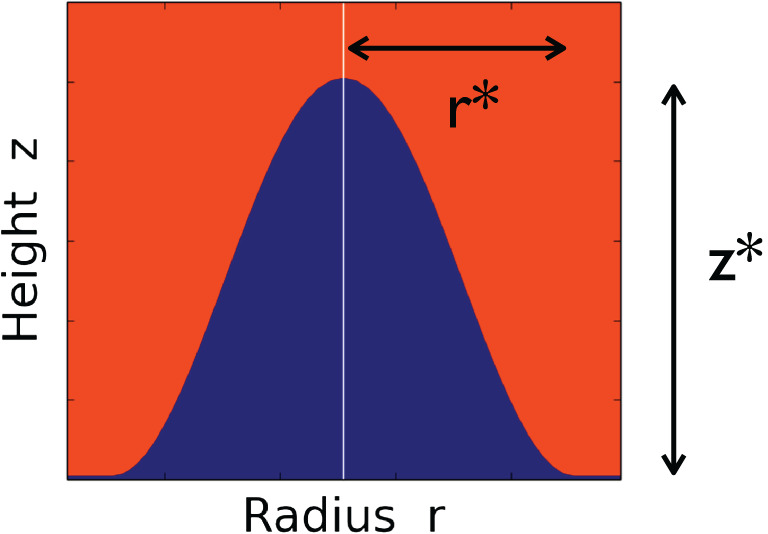
Initial configuration of the cold pool in the idealized LES setup, showing a cross section through the cold air anomaly of radius $r^{*}$ and height $z^{*}$.

To test the behavior of the tracers in a simple flow, the tracking is applied to an idealized CP that spreads into an environment approximately at rest, simulated in a high‐resolution LES setup (Figure 6). The CP is initialized as a “mountain” of cold air (Appendix A and Figure A1). The tracers are emitted along a circle around the center of the initial cold air anomaly at the beginning of the simulation, at a radius slightly larger than the anomaly. After initialization, the cold air anomaly collapses and spreads isotropically as a CP. Considering azimuthally averaged radial (Figure 5a) and vertical velocities (Figure 5b) at different times after CP initiation, the CP's vortex ring is clearly visible as a peak in horizontal velocity and a dipole in vertical velocity. Conversely, the CP edge (gust front) is identifiable as a peak in convergence, that is, the maximum gradient in $v_{r}$, which, by continuity, coincides with a peak in updraft speed w (Figures 5a and 5b, blue triangles). When the cold air front reaches the tracers, they are advected by the generated velocity field and trapped ahead of the narrow band of maximum horizontal convergence (Figures 5a and 5b, black dots). The tracers are advected using the velocity fields at an output rate of Δt=100 s without using sub–time steps ( n=1 in equation (5)), which proves to give the best results for the narrow structure of the CP gust front that is characterized by sharp gradients in the velocity field (Figure 5).

Qualitatively similar structures can be observed for a single CP from the UCLA simulations (cf. Figure 10), where the CP gust front spreads more slowly than in the idealized case and is much wider (Figure 5c). The larger gust front width is likely due to the coarser model resolution and a forcing, namely rain reevaporation, that persists beyond the time of initialization. Due to the lack of circular symmetry of this CP, the mean radius of the convergence zone is not as well defined, but one can nonetheless see why the method works: even though tracers are emitted behind the CP gust front (at values of r closer to the the CP center than the peak in radial velocity), they continuously travel toward the CP gust front and eventually reach it. Indeed, one can argue that the method robustly identifies the gust front, as long as tracers are emitted either at locations where the radial velocity is larger than that at which the  gust front propagates (tracers will then “catch up”) or at locations of larger r than that of the gust front (the gust front will “catch up” with the tracers). Inspecting again the radial velocity fields (Figures 5a and 5c), it is seen that the speed of the front itself is far smaller than the radial velocity at smaller values of r. Hence, the locus of the gust front, where radial velocities are defined to decrease rapidly, constitutes a type of fixed point in the space of radial velocity: Slight fluctuations of radial tracer position will always be balanced out by the negative velocity feedback associated with these departures.

Returning to the case of the idealized CP (Figures 5a and 5b), it is found that the near‐perfect circular symmetry is preserved during the first 30 min after initiation and the horizontal velocity is oriented almost exclusively in the radial direction. During this time interval, the tracers are homogeneously distributed along the CP front (Figures 6a, 6b and 6d, 6e). At a more mature stage, the circular symmetry is broken by the development of so‐called “lobe‐and‐cleft” instabilities along the gust front (Wakimoto, 2001) (Figure 6c and 6f). Around the protruding lobes, the speed tangential to the CP front is increased, leading to the accumulation of tracers at the troughs (clefts), while the leading edge of the lobes is depleted from tracers. This inhomogeneous tracer distribution is reflected in the average tracer radius appearing smaller than the average radius of the convergence zone (steepest gradient in velocity) for t≥2,000 s (Figure 5b). The areas of increased tracer concentration mark locations of horizontal convergence of tangential and radial velocity, which coincide with regions of strong vertical velocities and thus indicate points of increased likelihood of triggering new convection events. Thus, the tracking allows studying the detailed structure of CP instabilities which can be important for the triggering of convective events and CP collisions.

## 4 Application of Particle‐Based CP Tracking

**Figure 7:**
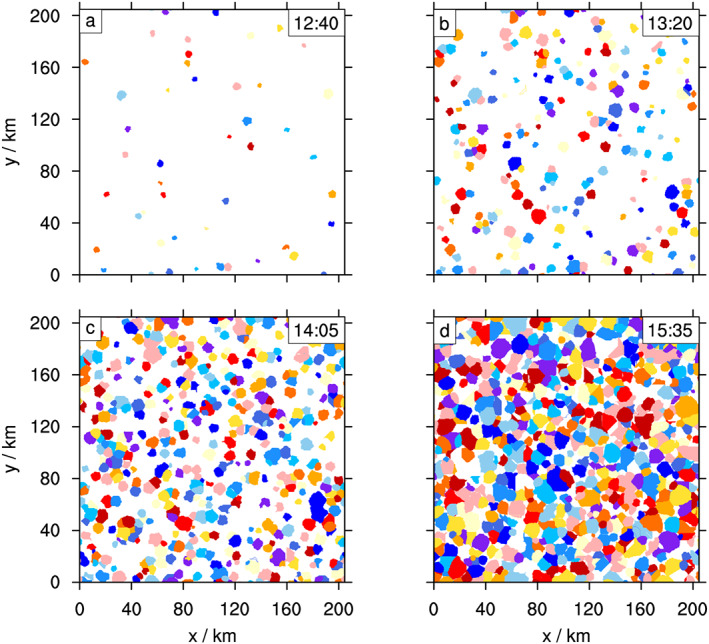
Cold pool objects throughout the model day. Identified CPs at different development stages within the diurnal cycle simulation (CTRL simulation), times within the model day as labeled in each panel. A patch of any given color represents the area enclosed by a CP gust front. Colors are chosen at random and have no meaning other than distinguishing CPs. (a) Initial deep convective cells. (b) Some CPs collide and clump together. (c) CPs start to fill the model domain (percolate) and overrun other CPs. (d) CPs nearly cover the entire domain area.

The pronounced diurnal cycle in the simulations sets off many convective cells within a short period of the model day, and the cells eventually occupy the entire model domain, with temporally changing characteristics (Moseley et al., 2019). Such nonstationarity allows for testing of the tracking for its robustness under varying boundary conditions. The increasing CP area becomes apparent upon inspection of the CP objects, detected by the current method at different times of the model day (Figure 7).

### 4.1 Identification of Convergence Highlighting CPs

**Figure 8:**
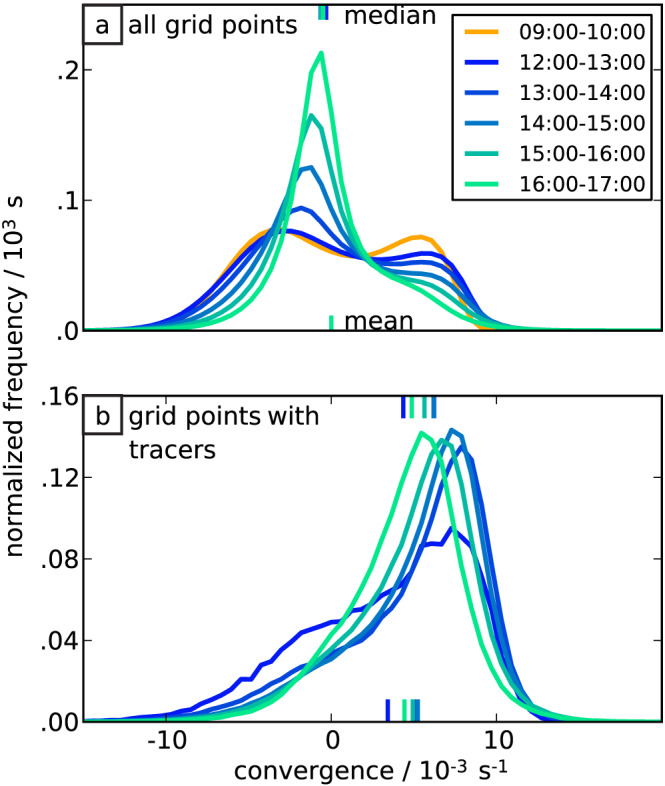
Horizontal convergence conditional on the presence of tracers. Normalized probability density functions of convergence at z=50 m (lowest model level) for (a) all grid cells in the model domain, (b) grid cells occupied by one or multiple tracers. Each curve represents one time interval within the model day (as marked in the legend).

As discussed (section 3.7), the tracer method should cause tracers to be advected into the typically narrow band of strong convergence separating the CP from its surroundings. To check this, we distinguish three qualitatively different stages within the diurnal cycle: (a) In the late morning (9:00–10:00 hr), the convergence pattern corresponds to a Rayleigh‐Bénard‐type circulation (Haerter et al., 2017; Moseley et al., 2016), formed by shallow cumulus convection with light drizzle, which generates very weak CPs (Glassmeier & Feingold, 2017). These weak, less disruptive, CPs are not discussed in the present study. At this time, convergence is compensated by divergence of similar magnitude, as is evident from the bimodal and approximately symmetric probability distribution of convergence within the lowest model level (Figure 8a). (b) Around noon (12:00 UTC), convection becomes deep and generates CPs with pronounced convergence bands. The stronger contrast between convergence and divergence is reflected in the elongated positive tail marking the occurrence of strong convergence (near $10\times 10^{-3}$ s ${}^{-1}$) (Figure 8a). (c) In the afternoon (after 14 hr), the number of precipitation cells reduces. CPs can now expand further before they collide with others and thus areas of weak divergence dominate the probability distribution. The number of active precipitation cells gradually decreases, whereas their typical intensities continue to increase (Haerter et al., 2017), causing more intense convergence values.

The convergence bands enclosing the CPs can be analyzed by conditional statistics taken over grid cells occupied by tracers (Figure 8b). The distribution of this subset is positively skewed and peaks at positive convergence values. The peak roughly coincides with the second peak in Figure 8a, proving that the majority of tracers occupies cells with strong positive convergence and identifies the CPs based on the strong convergence at their leading edge. Only a small fraction of these tracers occupies cells with divergence, mainly in the early hours of the deep convective regime.

There are multiple reasons why many grid cells with convergence remain unoccupied: At an early stage, some belong to drizzling shallow convective cells that do not enclose deep convective CPs and thus are not tracked (Figure 2). At a later stage, the convergence bands become wider and tracers may not cover the entire width of the bands. In addition, the tangential movement of tracers along the CP boundaries causes tracers to accumulate in only a small number of grid cells (*Details*: section 4.3).

### 4.2 CP Population

**Figure 9:**
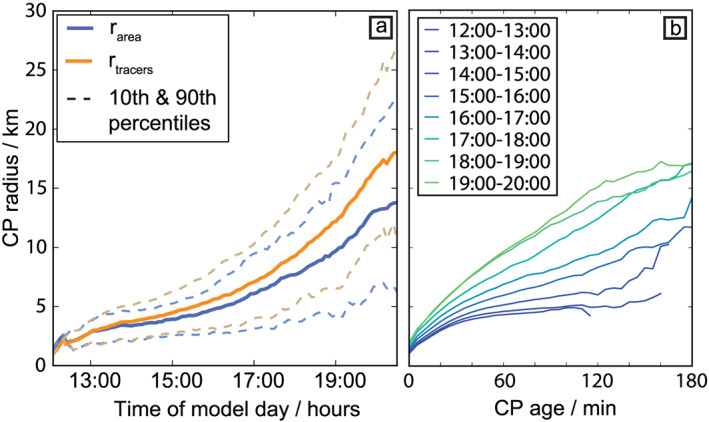
Dynamics of cold pool radius. (a) CP radius versus the time of day (hours), computed from the mean distance between the tracer particles and the CP center (solid orange) and from the area of the CP objects (solid blue). The 10th and 90th percentiles are indicated in dashed lines of corresponding similar colors. (b) CP radius versus CP age at different time intervals during the model day (as labeled in the panel). These periods were derived by binning all CPs in 1‐hr intervals according to the respective times of tracer particle emission.

The CP tracking identifies convergence bands and assigns them to corresponding CPs and parent precipitation events. This allows to track CPs as individual objects and to analyze the distribution of CP properties beyond the domain mean behavior including conditional and higher‐order statistics (Figure 9). The CP radius can either be retrieved from the mean distance of tracers to the CP center or by the CP area (Figure 9a). The mean distance of all associated tracers to the corresponding CP center provides a direct measure of CP radius $\left\langle r_{\text{tracers}}\right\rangle$. By assuming a CP to be spherical, an effective radius $r_{\text{area}}$ can be retrieved from the area given by the CP object, which results in slightly lower values than the former method. Note also that the spread, illustrated by the percentiles, increases during the day, indicating that CPs at this stage vary strongly in size.

The increase in average spatial scale with time (Figures 9a and 9b) is affected by different development stages of the deep convective CPs over the course of the diurnal cycle. In the first hour after the onset of heavy precipitation from deep convection (12:00–13:00 hr), only a small number of deep convective CPs emerges (Figure 7a). These CPs can expand without collisions to an average effective radius of 5 km, which is reached after ∼50 min (Figure 9b). The number of new CPs forming increases strongly in the afternoon, where the majority of CPs forms between 14:00 and 16:00. During the early afternoon, the wide spread in CP size can be explained by the coexistence of mature and young CPs. However, after the termination of convection, the continued increase in spread of CP size (Figure 9a) indicates that nearly the entire model domain is covered by CPs (Figure 7d) and many CPs interact with other CPs, preventing them from spreading beyond a certain radius.

### 4.3 Tracer Specific Analysis

**Figure 10:**
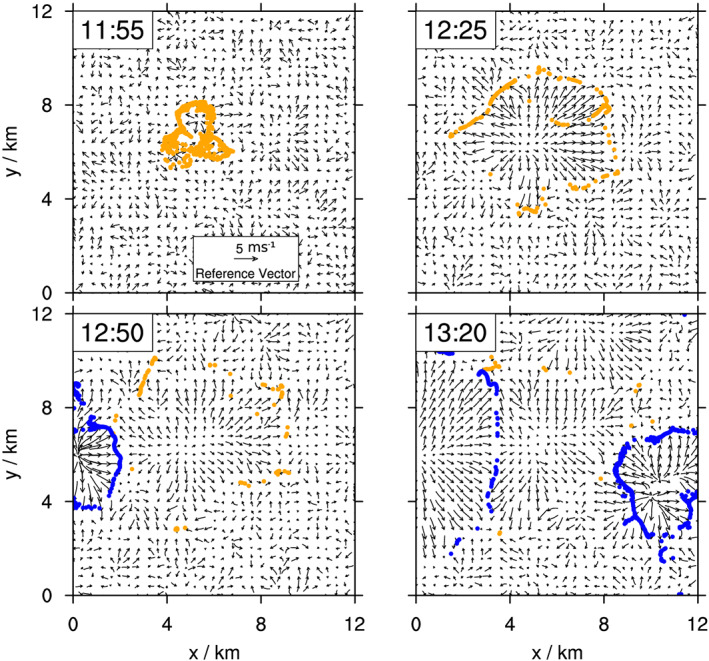
Tracer accumulation through tangential advection. Horizontal wind field and tracer position for first CP (orange points) and neighboring CPs (blue points) at different times showing tracer clumping during CP expansion.

In contrast to an object‐based method, the Lagrangian approach taken here, provides local information of the circulation at the CP gust fronts. Tracer dynamics allows for the analysis of the explicit circulation parallel to the CP boundaries. The distribution and accumulation of tracers over grid cells along the CP boundary is a measure of how homogeneously tracers spread along the expanding CP. Figure 10 provides an example of tracers accumulating in certain grid cells under the action of the horizontal wind field. We break the velocity vector of each tracer particle down into a radial and a tangential component and consider the time dependence of these two components (Figure 4). The plot shows a marked increase in $v_{t}$ after only ∼3 min, compared to the idealized CP (section 3.7), where tracers were found to maintain a nearly circular shape for as long as 30 min. This comparison indicates, that the environment might have a systematic impact on the tangential fluxes along the CP gust front. Causes for tangential tracer redistribution might be CP collisions and the wind field generated by CPs and convection.

**Figure 11:**
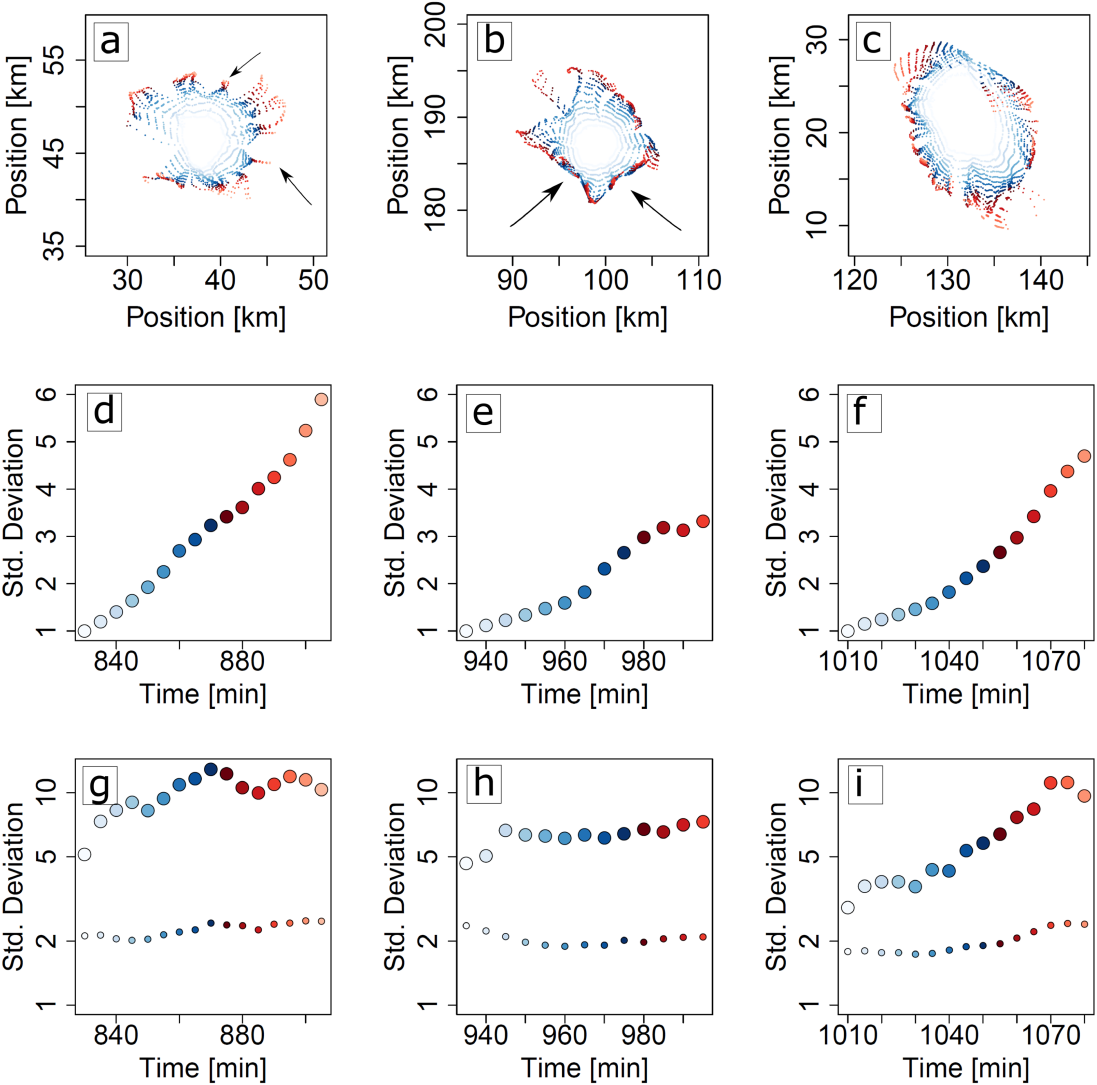
Tracer accumulation during cold pool expansion. (a–c) Examples of cold pool gust fronts as they spread during their life cycles. Colors from blue to red indicate times in steps of 5 min relative to the initial detection of each cold pool. Note the near‐isotropic gust front spreading at early times, but the inhomogeneous shape at later times. (d–f) Time series corresponding to (a)–(c), respectively, showing normalized standard deviation of the azimuthal distance for each cold pool gust front, computed by ${\left(\mathrm{var}(\Delta \phi ,t)/\mathrm{var}(\Delta \phi ,t_{0})\right)}^{1/2}$, where Δϕ is the azimuthal distance between any two neighboring tracers and ϕ≡arctan(y/x), with x and y the two horizontal coordinates of each tracer, and $t_{0}$ is the time of initial CP detection. Points of each time series are colored to match the colors of each gust front in the top row. (g–i) Analogous to (d)–(f), but derived from tracer multiplicities per gridbox (large circles) as well as tracer multiplicities, when distributing these at random into the same gridboxes (small circles).

To explore such tangential tracer transport in more detail, consider several examples of CP gust fronts, tracked at different times within the model day (Figures 11a–11c). At the initial stages of CP expansion (blue shades), gust front spreading is nearly isotropic, with near‐circular symmetry of the pattern formed by the tracer particles. At a later stage, ∼20 min after initial CP detection, the gust front contour looses its symmetry and fragments into various subsections. In each of these subsection, the number concentration of tracer particles is much larger. In some cases (panel a, exemplifying the early stage of the diurnal cycle), this fragmentation appears to occur without obvious impact of individual surrounding CPs, and a large number ( ∼10) of locations, where tracers accumulate, can be distinguished (note the arrows). We hypothesize that this symmetry breaking might again be due to a lobe‐and‐cleft instability (cf. Figures 6c and 6f). In other cases (panel b), the initial symmetry is reduced by a combination of small‐scale “sinks” and larger‐scale, near‐linear fronts. Two of these frontlines, reminiscent of the network formed in Voronoi diagrams (Haerter et al., 2019), are highlighted by arrows.

To quantify the decrease of symmetry, we first consider only the azimuthal component of each tracer at each time step of the CPs shown in Figure 11. We first sort all tracer particles by their azimuth and evaluate the angular distance between any two azimuthal neighbors. We then compute the standard deviation of these nearest‐neighbor azimuthal distances and normalize to the initial standard deviation, at the time when the corresponding CP was first detected (Figures 11d–11f). For the three examples shown, the standard deviation increases systematically with time. However, in the case of the early‐stage CP (panels a and d), the increase in standard deviation proceeds at a near‐constant slope, whereas in the case of the later stage CP (panels b and e) a relatively abrupt increase in standard deviation occurs after ∼20 min. We attribute this increase to a collision event with surrounding CPs, which falls in line with the relatively straight “gust front lines,” highlighted in panel b.

As an indicator of horizontal convergence it is useful to quantify, how strongly tracer particles are concentrated within a confined area. To this end, we consider the loci of all tracers of a given CP (now using both horizontal coordinates) and record the number of tracers in any model grid box at any time. We then distribute the same number of tracers at random over these gridboxes and compare the the distributions of the simulated versus random tracers regarding their standard deviation. The comparison shows, that the simulated distribution yields significantly larger standard deviation ( Figures 11g–11i), indicating that tracers become more concentrated than would be expected from a random (binomial) process.

**Figure 12:**
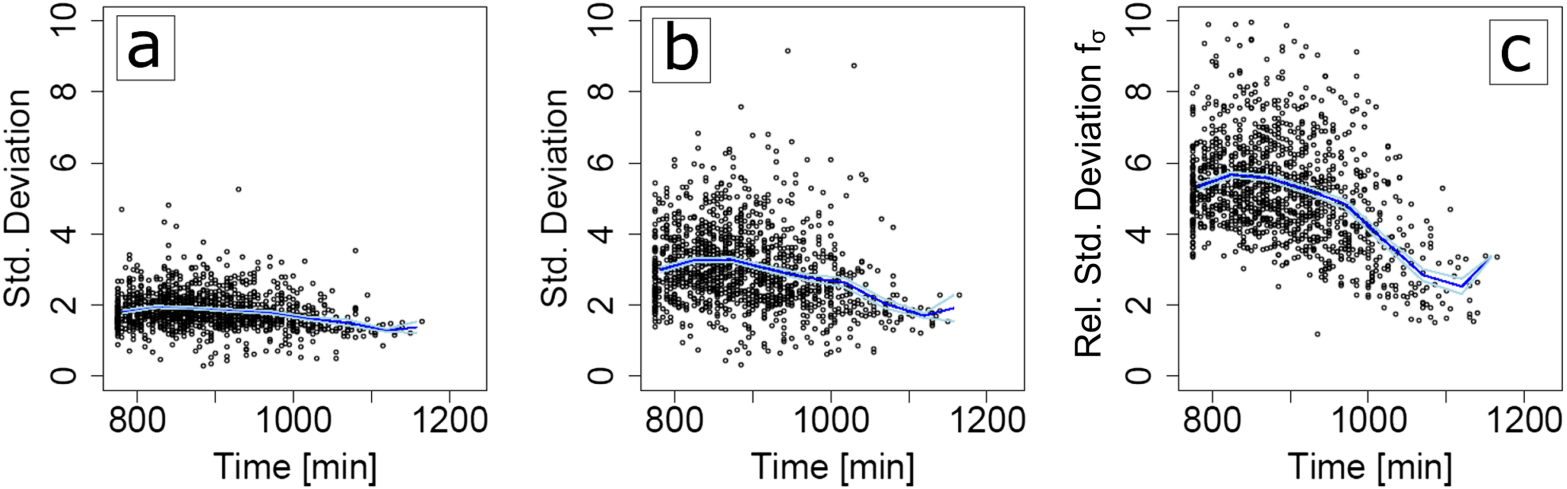
Diurnal time dependence of tracer accumulation. (a) Normalized standard deviation of the azimuthal distance between tracer positions after 25 min, evaluated for all CP gust fronts with lifetimes of at least 50 min. Dark and light blue curves indicate the mean and standard error, computed over 25‐min time bins. (b) Similar to (a), but after 50 min. (c) Relative increase of standard deviation after 50 min, when comparing the actual multiplicities of tracers to a random distribution for the same number of gridboxes (cf. Figures 11g–11i). The standard deviation in (c) is taken relative to the randomized counterpart (e.g., in the examples in Figure 11g‐i, the ratio of large and small points, 50 min after CP initialization).

To study the dependence of tracer accumulation on the diurnal cycle, we now repeat the analysis of the azimuthal nearest‐neighbour distance for each timestep in the simulation, using all available CP gust fronts of a given age (Figure 12a, b). The number of tracers per model gridbox for any CP at age 50min is again compared to the random distribution of the same number of tracers and occupied gridboxes (*compare:* Figure 11g–i), defining the relative standard deviation f = var(n; t = 50min)=var_rand_(n; t = 50min) (Figure 12c). We find that tracer accumulation occurs during all times of the diurnal cycle, indicated by the increasing standard deviation of azimuthal distance with CP age and a relative standard deviation *f* larger than one throughout the whole simulation time window where CPs are identified.. However, at later times in the CP life cycle, the accumulation appears to be somewhat slower. We attribute this to the gradual increase of spatial scales and the reduced impact of collisions at earlier times of CP expansion.

**Figure 13:**
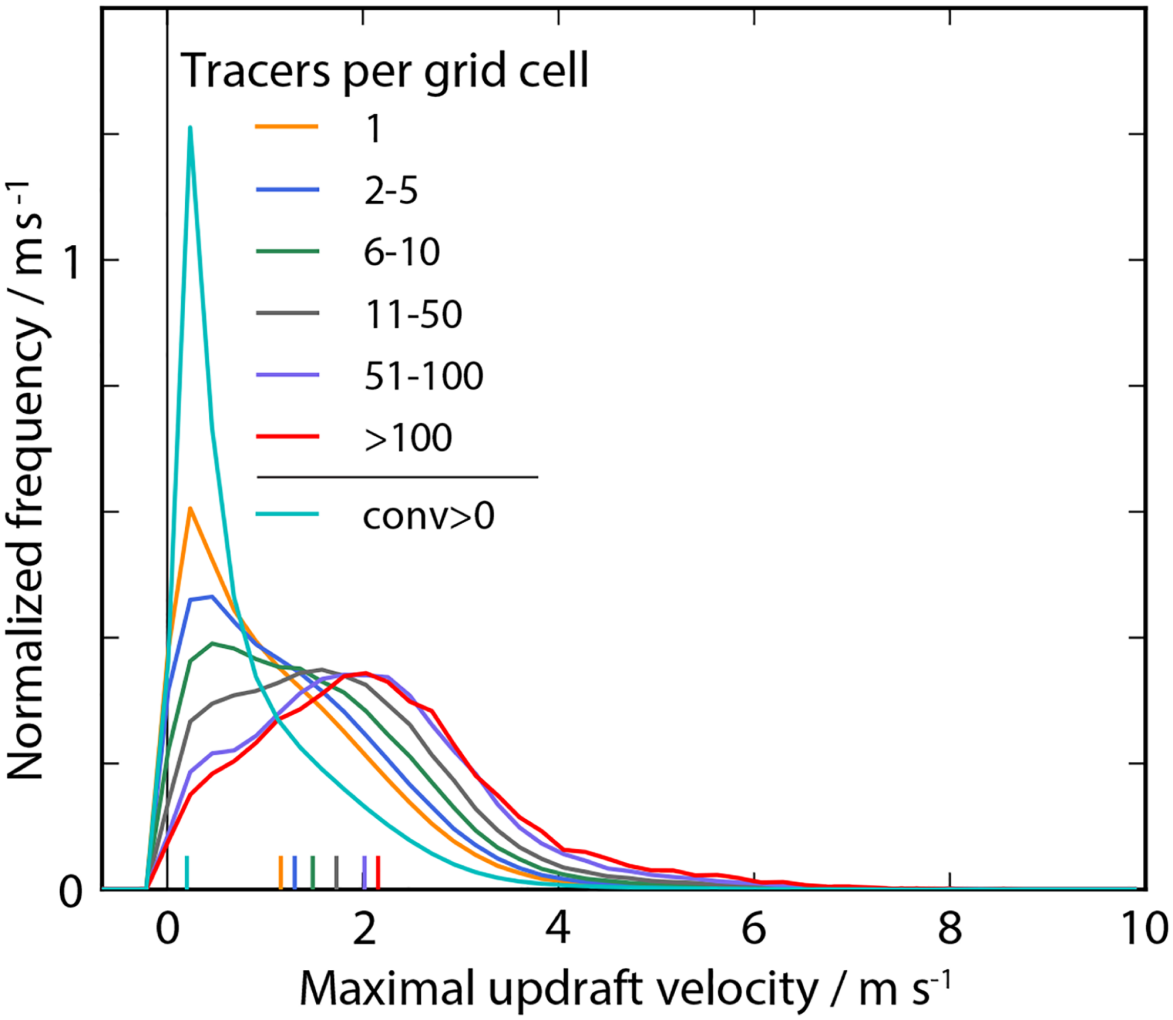
Quantifying tracer accumulation. Distribution of maximal updraft velocities in the lowest 20 model levels ( z<2.1 km) conditional on grid cells with the specified numbers of accumulated tracers (legend) as well as for all grid cells with positive horizontal convergence (conv > 0). Mean values are illustrated by small vertical lines in corresponding colors near the horizontal coordinate axis.

Can larger numbers of accumulated tracers predict convection triggering? An important indicator for convection are updraft velocities. We therefore now condition our tracer data on grid cells with varying tracer occupation (Figure 13). It is found that the larger the number of tracers within a given gridbox, the larger the maximal updraft velocities in the lowest 20 levels over these cells. The same holds true for the likelihood of strong updrafts as well as the average updraft velocity. When conditioning on gridboxes with convergence, the frequency distribution falls off more quickly than any of the distributions for gridboxes with tracers. These findings suggest, that a detailed study of tracer accumulation can give a robust indication of updraft potential.

## 5 Conclusion and Outlook

This study introduced a simple method to track CP gust fronts in the network formed by the narrow near‐surface convergence bands in high‐resolution simulation output. The method identifies each area enclosed by such a convergence band as a CP and associates it with its corresponding precipitation event that generated it, termed “parent precipitation events.” Exploiting the flow patterns of these identified CPs, combined with the generated horizontal convergence, any passive tracer, released at an appropriate time near the boundary of a precipitation cell, will inevitably be transported to the corresponding gust front and remain there until the front decays. The algorithm attributes to each tracer particle an identifier that relates it to its respective parent precipitation event.

The tracking is simple, as it requires only three two‐dimensional fields: surface precipitation to initially identify a potential CP as well as the horizontal wind field ( u and v at the lowest model level) to calculate the trajectory for every tracer. As a criterion for tracer emission we use the horizontal divergence field, retrieved from the horizontal wind field. This criterion improves the tracking, because tracers are found to be transported to the edges of the CP in question, whereas without the criterion the prevailing wind field often causes tracers to be advected asymmetrically to one side of the rain event and its CP. We implement the criterion, by requiring a lower threshold on divergence, $D_{0}$. This allows to define the appropriate timing of when to initialize the CP track after the onset of precipitation. We verify by using conditional probability density functions, that tracer positions indeed coincide well with the band of near‐surface convergence and follow the horizontal low‐level wind field.

The tracking is fairly robust to assumptions made on the parameters (Table 1), as the tracking results only indirectly depend on these: The CP outlines are determined by the advection of tracers, and the parameters only serve to set reasonable starting conditions for the tracers. The tracers and their trajectories finally carry all information about the CP boundary, including the CP area, wind speed at the edges, and the CP propagation speed. A CP is terminated by a threshold criterion for the relative change of CP area, thus making the termination of CPs independent of the absolute CP area, lifetime, or speed of spreading. CPs terminate when they no longer spread and are compressed by surrounding air masses, such as expanding CPs in the neighborhood.

The tracking method was successfully applied on a LES data set of continental deep convection over the course of a diurnal cycle. These simulations bring about a wide range of CPs characteristics: from relatively small CPs in the morning hours that can spread freely to large CPs in the afternoon, for which spreading is often hampered by the interaction with other CPs. Though interacting and overlapping CPs challenge the tracking, the tracking identified most CPs as an individual object. At late afternoon hours, CPs in their early development can be affected by the background wind field formed by older, similarly intense CPs. Tracking these CPs was made possible by using the divergence field as additionally constraint on tracer emission. The method allows for the tracking of these relatively persistent CPs over their entire lifetime, because the tracer tracking is not affected by dissipating properties such as the temperature depression nor does it impose an absolute cutoff on lifetime. A caveat, which could be improved in an extension, is that emitted tracers typically require several time steps before reaching the gust front.

It was tested qualitatively, that even in simulations with weak wind shear the tracking captures the majority of CPs without adjusting the input parameters. However, as in the presence of wind shear CPs often appear as an open ring, the tracking mainly identifies the convergence of the windward side of the CP.

In addition to identifying and tracking the CP area, as other existing CP tracking schemes are capable of (Drager & van den Heever, 2017; Schlemmer & Hohenegger, 2016; Torri et al., 2015), the particle‐based method is able to give more detailed information about spatial variations along the CP gust front. This is done by tracking the spatial distribution of the tracers, revealing that many grid cells along the CP gust front remain unoccupied. This is a consequence of CPs not simply spreading radially away from the precipitation center, but velocities tangentially to the spreading direction acting to accumulate tracers in only very few grid cells. This redistribution of tracers differs strongly from a random distribution and provides information on locations of strong updrafts, where new convection might be triggered.

The choice of two‐dimensional tracers was made with a clear goal in mind: the detection of the CP outline as defined by the convergence band. If tracers are seeded at low levels and advected using the three‐dimensional velocity field, they may occasionally also be advected into the convergence zone. However, given that they reach the gust front, they are inevitably transported upward to higher levels of the boundary layer or occasionally return into the CP through the wake behind the CP edge. For these reasons, the concentration of tracers remaining at the gust front will be much lower and the signal indicating the gust front position will be washed out. Additionally, to reach the same association between rain events and their gust fronts, new tracers would repeatedly need to be seeded near the surface and a tracer lifetime would have to be defined to remove the tracers that have been mixed into the environment and deteriorate the gust front statistics.

On the other hand, three‐dimensional tracers may allow for a more complete picture of the three‐dimensional circulation of the CP, the history of its air parcels in a Lagrangian sense and its effects on environmental air parcels (Torri & Kuang, 2016, 2019), which is not the focus of the method described here. One may also argue that if we are interested in the convective triggering by CPs, the three‐dimensional structure, the height and air properties of the forced updrafts are important to be detected. An extension of the method described here could however exploit the knowledge we obtain on loci of strong updraft origins (Figure 13). Assuming the updrafts to be coherent vertical structures (Lenschow & Stephens, 1980) still allows to infer information about locations of increased triggering likelihood.

Strong horizontal convergence, that is, strong variations in horizontal velocities, can further indicate the boundary between regions of differential surface fluxes: It is well‐known that strong horizontal winds under CPs enhance surface fluxes and thus lead to a moistening of the atmosphere, causing favorable thermodynamic conditions for subsequent convection (Tompkins, 2001a). The enhanced surface fluxes, combined with updrafts generated in the convergence band, can help moisture accumulate in this region—locally increasing the potential for deep convection (Tompkins, 2001a). Thus, exactly the narrow bands of strong convergence are important to consider as CP boundaries in terms of their ability to trigger new convection. Whereas CPs are known to trigger new convection, a future goal, which could be accomplished using the present tracking method, is to identify precisely at which locations *along* their gust fronts and at which times new convection is triggered.

As mentioned, the particle‐based CP tracking was here applied on simulations with strong diurnal variations. We expect that the tracking will hence also be suitable for simulations with less variable boundary conditions, where CP variations are weaker and fewer challenges are posed due to their changing characteristics. Such simulations could include those resulting from radiative convective equilibrium boundary conditions, where CP properties typically vary less. Also there, the direct causal effect of one or several CPs on subsequent convective precipitation cells is far from settled—yet it is relevant for disentangling the role of CP interaction in emergent system‐scale phenomena, such as convective self‐aggregation.

## Appendix A Model Description (Proof of Concept)

### A.1

The idealized CP simulation is run using the LES model PyCLES (Pressel et al., 2015), which equivalently to the UCLA‐LES solves the anelastic equations and represents subgrid scale diffusion using the Smagorinsky scheme (Smagorinsky, 1963). The two models differ from each other primarily in the thermodynamic prognostic variable, where PyCLES uses moist entropy instead of liquid water potential temperature. The simulation is run at isotropic resolution of Δ=50 m in a domain of horizontal extension 20 km and height of 12 km, employing periodic lateral boundary conditions. Numerical integration is employed for 1 hr, using an adaptive time step on the order of 1 s. No surface fluxes of heat and momentum are applied, leading to a longer lifetime of the CP. Turbulent fluctuations are initialized by adding noise in potential temperature of amplitude $\theta^{\prime }=0.05$ K in the lowest levels ( $z\leq z^{*}+2\Delta$), where $z^{*}$ is the height of the initial cold air anomaly.

The CP is initialized as a“mountain” of cold air anomaly ( θ=297 K) in a neutrally stratified and dry environment of $\theta_{0}=300$ K (Figure A1). The anomaly, centered at the origin, is bounded by a cos ${}^{2}$‐envelope of radius $r^{*}=1$,000 m and height $z^{*}=1$,000 m,

(A1) $$z_{\max}(r)=z^{*}\cdot \cos^{2}\left[\frac{\pi }{2}\frac{r}{r^{*}}\right],\;0<r\leq r^{*}$$

with $r=\sqrt{x^{2}+y^{2}}$. To lower numerical errors, a transition layer of 200 m width is applied, in which the temperature anomaly decreases smoothly to zero. When the simulation is started, the mountain collapses and the cold air spreads isotropically along the surface, forming a CP.
